# Effective population size and N_e_/N ratio for two populations of Northern chamois (*Rupicapra rupicapra*) on the edge of the species’ distribution range

**DOI:** 10.1186/s12862-026-02543-7

**Published:** 2026-06-26

**Authors:** Susanne Jacobs, Hendrik Edelhoff, Cornelia Ebert, Wibke Peters

**Affiliations:** 1 Wildlife Biology and Management Research Unit, Bavarian State Institute of Forestry, Freising, Germany; 2 Department Wildlife Genetics, Seq-IT GmbH & Co.KG, Kaiserslautern, Germany; 3 Wildlife Biology and Management Unit, Technical University of Munich, Freising, Germany

**Keywords:** Effective population size, N_e_/N, *Rupicapra rupicapra*, Microsatellites, Population size

## Abstract

**Background:**

Effective population size (N_e_) is a fundamental parameter in conservation genetics, providing relevant information on the adaptability and long-term viability of populations. In contrast, wildlife management strategies, particularly for hunted species, often rely on estimates of abundance (N) without incorporating genetic data. Accounting for N_e_ in wildlife management concepts could add important information on population viability, especially for species that are regulated mainly by humans. The iconic mountain dwelling chamois *(Rupicapra rupicapra*) is widespread throughout the Alps, and many populations are influenced by human activities, including hunting.

**Results:**

We derived N_e_ using genetic data from 16 microsatellite loci for two chamois populations in the Bavarian Alps, Germany. Estimates of N based on Bayesian spatial capture-recapture (SCR) from a previous study allowed us to calculate the N_e_/N ratio. N_e_ values per generation in the two study areas were 514 (CI 342–949) and 179 (CI 129–266). Estimated median N_e_/N ratios were similar in both study areas but had large confidence intervals: 0.56 (CI 0.34–0.96) and 0.58 (CI 0.39–0.88). Confidence intervals of N_e_ estimates varied between the two study areas, and sample size had a substantial influence on the precision of the estimates, as expected from previous studies. We discuss the challenges in obtaining robust N_e_/N estimates, which are crucial when N_e_ and N are applied for conservation purposes.

**Conclusion:**

N_e_/N ratios were similar between the two study areas, but confidence intervals were wide, reflecting substantial uncertainty. Even with extensive data, accurately estimating these ratios remains challenging, highlighting potential limitations for their use in population monitoring and management.

**Supplementary Information:**

The online version contains supplementary material available at 10.1186/s12862-026-02543-7.

## Background

The effective size of a population (N_e_) is an important parameter in population, evolutionary and conservation genetics. This parameter determines the amount of genetic variation that is lost through genetic drift and inbreeding and has been widely used to assess and monitor the population status of endangered species [9, 23]. More recently, N_e_ has been proposed as an essential biodiversity variable (EBV; [30]) that can be used in monitoring programs aimed at conserving genetic diversity, one of the three basic elements of biodiversity [48]. Finally, the proportion of populations with N_e_ > 500 was adopted as a headline indicator in the Kunming–Montreal Global Biodiversity Framework (CBD/COP/DEC/15/5). Now that genetic EBVs have been established and N_e_ is applied as an indicator for threats of genetic diversity loss, genetic data has become a more relevant factor in biodiversity assessments.

In the absence of comprehensive genetic data, N_e_ is often estimated from estimates of N, the total population size, and assumptions about the N_e_/N ratio. To date, precise estimates of abundance are challenging to obtain and may require substantial effort [18, 40]. If N_e_/N is not known for a particular species, standard values—such as 0.1—can be used to estimate N_e_ from an estimate of N, contributing to the imprecision of the measure. Species-specific N_e_/N values can prevent overly conservative N_e_ estimates [31] and therefore contribute to a more accurate identification of populations with conservation concern.

While N_e_ describes genetic processes, it is shaped by underlying demographic factors. When deriving N_e_ from these factors, detailed demographic data are required; however, key variables – such as total population size/abundance (N), sex ratio, family size or variance in reproductive success – are difficult to obtain in wildlife populations [64]. Therefore, estimates of N_e_ based on genetic marker data have become standard, promoted by increased availability and affordability of genetic data and methods [41]. Concurrently, the accuracy of N_e_ estimates under varying conditions in nonideal populations has been extensively studied with both simulated and real world populations, creating a robust framework for the estimation of N_e_ based on genetic data (reviewed in [70]). Despite a wealth of knowledge on the estimation of N_e_ based on genetic data, violation of assumptions remains a challenge for deriving genetic N_e_ estimates in wildlife populations: generational overlap, population structure, migration, biased sampling and any combination of these may lead to biased estimates that are unpredictable regarding their direction and magnitude [27, 61, 67, 70]. Additionally, small sample sizes can affect analyses, leading to over- or underestimation of N_e_, large confidence intervals or infinite values [3, 42, 51, 57].

The N_e_/N ratio itself also provides information on parameters relevant for conservation [22, 23, 37, 51]. Specifically, comparing simultaneous estimates of N and N_e_ can offer important insights into changes in life-history parameters (e.g., variation in reproductive success) or fluctuations in population size of the studied population or species [51, 71]. Due to the absence of reliable data, opportunities for this have been rare to date. In addition to imprecision in estimates of N and N_e_, difficulties in calculating the N_e_/N ratio may arise from measures relating to different time spans, which is particularly important in species or populations with changing population sizes [45, 50, 54]. Therefore, for species of conservation concern, the ratio of N_e_ to N has been under debate, and estimates vary widely between but also within species depending on life history, population size or fluctuations thereof [10, 50, 71].

Here, we had the rare opportunity to build a case study on the comparison of N and N_e_ thanks to the availability of very detailed data for a species that has received much conservation and management attention in Central Europe. The Northern chamois (*Rupicapra rupicapra*) is a charismatic alpine ungulate species inhabiting mountainous regions in Europe and southwest Asia. The alpine subspecies *R. r. rupicapra* is distributed across the Alps, from France in the west to Slovenia in the east [11]. *R. r. rupicapra* is protected by European law through enlisting in Annex V of the Species and Habitats Directive. Thus, hunting is legal, but maintaining a favorable conservation status is required. The evaluation of population status is therefore highly relevant for wildlife managers. Throughout the Alpine Chamois range, monitoring usually comprises hunting bag data or various counting methods [13, 56].

Direct counts of chamois in open habitats are commonly conducted to detect population trends in this species. However, the detectability of chamois during direct population surveys varies considerably between habitats; hence, these types of surveys are limited to open areas, as individuals inhabiting forested areas cannot be counted [40]. Robust estimates of abundance and density across all landcover types can be obtained with non-invasive (e.g., genetic) spatial capture-recapture methods (SCR; [59]). These estimates are available for two intensively studied areas in the Bavarian Alps [19]. Furthermore, N_e_ has only been estimated for *R. r. balcanica* [43], and N_e_ and the N_e_/N ratio have been estimated for *R. r. tatrica* and an introduced population of *R. r. rupicapra* [78]. To our knowledge, the N_e_/N ratio has not been previously assessed for *R. r. rupicapra* in its native range.

Based on precise data on population size from the SCR study and tissue samples collected over multiple years for two study areas in the Bavarian Alps, we pursued two key aims: (a) to derive genetic effective population size for two chamois populations and (b) to compare N_e_ and N. First, we estimated N_e_ for chamois in two regions of the Bavarian Alps as an important measure of population viability and conservation status. We hypothesized that N_e_ would differ between the study areas because of strong density contrasts (chamois/100 ha) between both study areas. Next, we calculated the N_e_/N ratio for each study area. We expected N_e_/N values to be within the same order of magnitude in both study areas; however, we presumed that slight differences may have arisen due to past population bottlenecks or variation in age at first reproduction, maximum life expectancy, sex ratio or interindividual variability in reproductive success. In addition, we aimed to analyze the effect of sample size on the precision of N_e_ estimates to evaluate the sampling effort necessary when expanding the analysis to other areas or future monitoring.

## Methods

### Study areas and chamois populations

**Fig. 1:**
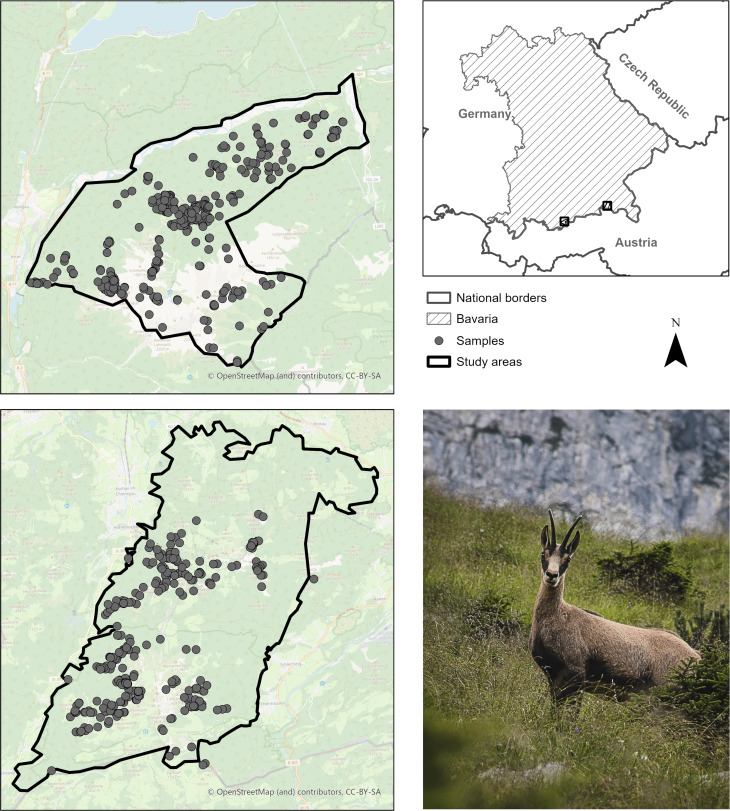
Maps of the study areas Karwendel (KW, top left) and Chiemgau (CG, bottom left). The gray circles represent the locations of samples within the study areas. The map on the top right shows the locations of both study areas in the German federal state of Bavaria. The photo shows Alpine chamois in the KW study area, photo credit: Juliane Warger. The maps were created in ArcGIS Pro using OpenStreetMap tiles as background (ODbL)

Genetic samples of chamois were obtained from two study areas in the Bavarian Alps, both of which neighbor Austria (Fig. 1). The study area “Karwendel” (KW) in the Karwendel mountain range covers approximately 5,250 ha of montane and subalpine habitats ranging from 800 to 2,350 m above sea level. The study area “Chiemgau” (CG) has a larger surface area (7,250 ha) but covers lower altitudes, with heights above sea level ranging from 600 to 1,800 m. Forested areas account for 70% of CG and 60% of KW. Outside of forests, alpine meadows and barren, rocky terrain are the prevailing habitats, with the latter being more pronounced in KW. The KW study area is completely protected as a nature reserve, while the CG area is partly protected. Yet, both areas are subject to a high level of anthropogenic influence—mainly through tourism, hunting and cattle grazing during the summer months.

Chamois abundance (N) was estimated by Edelhoff et al. [20] using feces that were systematically sampled during the fall of 2018 and subsequent SCR analyses. The estimated population sizes were 1,016 individuals (95% Bayesian credibility interval (CI) 935–1,105) for KW and 320 individuals (CI 264–393) for CG. The estimated sex ratio (male/female) was 1.0 in KW and 0.72 in CG. The main cause of mortality in both populations is assumed to be hunting. According to federal state law, chamois can be hunted from August 1 to December 15. Exceptions applied for designated areas to protect regenerating forests from browsing ungulates during the years considered in this study. The harvest density was 2.4 chamois/km² in KW and 1.6 chamois/km² in CG (average 2011–2018). Given the lower chamois density in CG, this results in a higher harvest rate in relation to population size. Natural mortality rates are not known, but we assume they are lower in CG and vary between years depending on habitat and weather conditions based on reports of local game managers.

### Sample collection and genetic analysis

We collected chamois tissue samples during the regular hunting seasons between 2016 and 2022. Samples were either obtained in the lab from lower jaws and kept frozen until analysis (hunting seasons 2016–2020) or from soft tissue directly collected by the hunters and preserved in 99% ethanol (2021 and 2022). For each harvested individual, date and location of hunting were recorded. In most cases, GPS coordinates were provided by the hunters; otherwise, individuals were assigned to a predefined hunting ground, resulting in a coarser spatial resolution. As N_e_ was estimated at the population level, fine-scale spatial resolution was not required for the analyses. Sampled chamois were aged by hunters by counting horn annuli [63]. Thus, we were able to determine the year of birth for most individuals present in the tissue sample by subtracting the age from the year the individual was hunted.

### DNA extraction and microsatellite genotyping

We extracted DNA from tissue samples using a commercial kit (Chemagic DNA Tissue Kit, Perkin Elmer, Rodgau, Germany) and a Chemagen MSM1 magnetic separator (Perkin Elmer, Rodgau, Germany). We selected 16 microsatellite markers already tested for chamois according to their variability and suitability for multiplexing and combined the markers in four separate multiplex PCRs (Table S1). These 16 microsatellites were amplified using the following PCR protocol: 95 °C for 15 min; 45 cycles of 94 °C for 30 s, 57 °C for 90 s, and 72 °C for 60 s; and 60 °C for 30 min. Amplification reactions were performed in a total volume of 12 µl via the Qiagen Multiplex PCR Kit (Qiagen, Hilden, Germany). The primers were used at concentrations ranging from 0.1 µM to 0.4 µM. We separated fluorescently labeled DNA fragments on an ABI3730 DNA analyzer and determined allele sizes using the ABI GS500LIZ size ladder (Applied Biosystems, Darmstadt, Germany). Allele calling was carried out using GeneMapper V.7 (Applied Biosystems, Darmstadt, Germany) and by manually checking all called alleles. We included two negative PCR controls in every PCR set to detect potential contamination. Each analysis was run in duplicates to minimize errors from amplification failure, allelic dropout, or false alleles. We deduced consensus genotypes from the duplicate results. Samples were typed as heterozygous at one locus if both alleles appeared twice, and as homozygous if all replicates presented the same result. We repeated the genotyping process another two times if results were ambiguous after the first two replicates.

### Markers and genetic diversity indices

All basic population statistics were conducted in the R environment (version 4.2.3). We formatted and handled genetic data with the adegenet package [34]. Loci were screened for null alleles using both the estimator of Chakraborty et al. [8] and Brookfield [7] implemented in the PopGenReport package [1]. We analyzed observed and expected heterozygosity (H_o_ and H_e_), coefficients of inbreeding (F_IS_), and allelic richness (A_r_) using the hierfstat package [28]. Deviations from Hardy‒Weinberg equilibrium (HWE) were estimated using the exact test from the pegas package [52] with 10,000 replicates of the Monte-Carlo procedure. We tested genotypic linkage disequilibrium (i.e., systematic deviations from linkage equilibrium across populations stemming from the physical linkage of markers) with the LD function of the genepop software [58], implemented in the corresponding R package. LD analyses were run with an MCMC dememorization of 10,000 for 1,000 batch runs of 10,000 iterations. We corrected for multiple comparisons by utilizing sequential Holm‒Bonferroni correction of p-values [32]. Genetic population structure can influence LD-N_e_ [74]; thus, we analyzed samples from both study areas with the STRUCTURE software ([55]; details in the supplementary material).

### N_e_ and N_e_/N estimates

We calculated values for linkage disequilibrium estimates of effective population size (LD-N_e_) with the NeEstimator 2.0 software [17]. This approach uses an unbiased estimator of Burrows’s Δ [72]. Following the recommendations of Waples and Do [73], we used a critical threshold for the lowest allele frequency (P_crit_) of 1/(2 S) for sample sizes n < = 25, 0.02 for 25 < n < = 100 and 0.01 for *n* > 100 to reduce bias while maximizing precision. We assumed random mating and obtained a 95% confidence interval with the jacknifing method. To analyze the potential effects of rare alleles that may harbor information on migration [74], we additionally ran all analyses with all P_crit_ values (1/(2 S), 0.01, 0.02, 0.05).

To calculate N_e_ per generation despite the age structure, we combined samples from consecutive cohorts corresponding to approximately one generation length, as proposed by Waples and Do [73], and further evaluated by Robinson and Moyer [57] and Waples et al. [76]. We used six years as the generation length in accordance with other genetic studies (6.24 years in Pérez et al. [53] and Leugger et al. [38], cited from Gaillard [24]. We selected the birth years from the interval with the largest sample size: 2015–2020. In addition, we calculated LD-N_e_ pooling all samples, including samples of unknown age. In the case of markers deviating from HWE or showing evidence of null alleles, N_e_ analyses were repeated, omitting the affected markers. Furthermore, we repeated the analyses excluding individuals identified as potential migrants based on STRUCTURE assignment probabilities.

As an alternative approach to estimating N_e_ in species with overlapping generations, we estimated N_b_, the effective number of breeders, and calculated N_e_ from this value [76]. N_b_ generally applies to a single cohort. However, owing to the small sample sizes of individual cohorts, we combined two cohorts each for the calculation of N_b_. As chamois usually give birth to only one kid per year and do not start reproduction until the age of two, we expect little bias and can rule out parent-offspring pairs in the sample [35, 76]. We selected the cohorts from 2018 to 2019 because parents of these cohorts are most likely to be included in the N estimate derived by SCR from feces collected during the fall of 2018; therefore, these cohorts are best suited for a direct comparison of N_e_ and N. To account for potential bias by age structure in populations with overlapping generations, we corrected the estimated N_b_ values with the two-trait adjustment by age at first reproduction (α) and adult life span (AL) proposed in Waples et al. [76]. We used α = 3 years and AL = 16 years (maximum age ω = 18 years). While some female chamois give birth at two years of age in favorable conditions [25, 60], many male chamois presumably do not start reproducing until they reach an older age [14, 26]. As recommended in Waples et al. [76] and Waples [69], we estimated α as the age at which we expect fecundity to be approximately 50% rather than the earliest age with fecundity > 0.

We calculated N_e_/N ratios considering both the credibility interval of the SCR estimates of N and the confidence interval of the LD-N_e_ estimate. In this study, N refers to the total population size, presumably also including kids and yearlings [20]. Uncertainty in the N_e_/N ratio was propagated using Monte-Carlo simulation. Because NeEstimator does not provide the jackknife sampling distribution, we approximated the uncertainty by simulating 10,000 values assuming a lognormal distribution parameterized from the reported 95% jackknife confidence intervals on the log scale. Posterior samples of census size were obtained from the SCR study by [20]. The N_e_/N ratio was calculated by dividing simulated N_e_ values by corresponding posterior draws of N. The resulting simulation-based distribution of N_e_/N was summarized using the median and 2.5% and 97.5% quantiles. LD-N_e_ estimates generally apply to the previous generation, as they measure the drift in the parent generation of the individuals sampled [45]. Comparing N_e_ and N from samples collected in the same period is feasible only under the assumption of a constant population size [50]. We assume stable population sizes on the basis of hunting bag data since the previous generation. In contrast, N_b_ applies to the parental generation that produced the offspring sampled. Thus, N_e_ estimates adjusted from N_b_ from cohorts more recent than the SCR study should be directly linked to N.

### Estimating sensitivity to sample size

To test how a reduction in sample size influences N_e_ estimates and their precision in the two study areas, we randomly chose subsets of 10, 20, …, 190, 200 individuals from the 2015–2020 generation sample. Subsampling was repeated 100 times per sample size category. We calculated the mean and harmonic mean for all sample size categories and assessed the percentage of infinite estimates. The relative root mean square error (RRMSE) was used to compare the precision of estimates with different sample sizes within and between study areas. Unlike simulation studies, our empirical study did not provide us with a definite “true” value of N_e_. Therefore, we chose the N_e_ estimate drawn from the full sample as the “true” value, assuming that this value is the most precise and accurate.

## Results

### Marker and basic population statistics

We obtained a total of 705 chamois samples (KW: 380, CG: 325), 376 and 311 samples with known ages for KW and CG, respectively. The sex ratio in the sample (males/females) was biased toward males in both study areas: 1.26:1 in KW and 1.56:1 in CG. All loci were polymorphic with a mean allele number of 9.25 +- 3.6 (8.3 ± 2.8 in KW; 7.5 ± 3.1 in CG). No evidence of null alleles was found for the CG study area (all 95% confidence intervals contained zero). Two loci, BM203 and OARFCB304, presented evidence of null alleles in the KW study area, however at low percentages (< 3%). We thus assumed no relevant effect of null alleles on downstream analysis, however, to rule out any potential bias, we repeated N_e_ analyses for KW excluding the two loci with weak evidence of null alleles.

Tests for departure from HWE were significant for two loci in CG after Holm–Bonferroni correction (OARFCB304, *p* = 0.02; INRA36, *p* = 0.03), but no significant deviation was detected in KW, suggesting no systematic deviation from HWE in specific loci. To rule out any potential bias by deviation from HWE on N_e_ estimates, we repeated N_e_ analyses for CG without the two loci. Linkage disequilibrium was significant after correcting for multiple tests for one locus combination in CG (BM203 and HEL1) but not in KW. We thus conclude that the observed LD is the effect of drift rather than physical linkage. LD by drift is the signal used for the estimation of LD-N_e_ in downstream analysis, and as such, significant deviations from linkage equilibrium are more likely to be observed with larger sample size to N_e_ ratios in finite populations [68]. Therefore, we included all loci in the N_e_ analysis.

Both allelic richness and heterozygosity were greater in KW than in GC, indicating a genetically more diverse population in KW. Small positive values of F_IS_ were detected for both study areas (Table 1).

**Table 1:** Genetic diversity measures for the two study areas

Population	*N*	A_*r*_	H_e_	H_o_	F_IS_
CG	325	7.5	0.64	0.63	0.014
KW	380	8.2	0.70	0.68	0.020

Analysis of population structure with the STRUCTURE software revealed a clear separation of the two study areas into two clusters (K = 2; Fig. S1 and S2). Within the KW study area, no genetic sub-structuring was detected with STRUCTURE (K = 1; Fig. S3 and S4). For the CG area, STRUCTURE results were inconclusive. The Evanno deltaK method revealed eight clusters, but the deltaK values were negligible (deltaK < 1.5), and the clusters did not show any spatial or temporal pattern, probably tracing back to fine-scale group structure (Fig. S5 and S6).

### Effective population size

As expected, the estimate of LD-N_e_ per generation calculated for the generation born 2015 to 2020 was greater for the KW study area, with 514 (CI 342–949) and 179 (CI 129–266) compared to the CG area (Table 2). Estimates excluding loci with slight evidence for null alleles (KW) or deviation from HWE (CG) did not strongly influence the results but led to a larger confidence interval in KW. Similarly, exclusion of individuals identified as potential migrants did not substantially change the N_e_ estimates (supplementary information, Table S4).

In CG, pooling all samples led to lower LD-N_e_ estimates, as expected from literature [57]. In KW, the pooled sample resulted in higher LD-N_e_ estimates (CG: N_e_ 148, CI 106–215; KW: N_e_ 549, CI 391–861). N_e_ calculated from N_b_ with the two-trait adjustment revealed N_e_ estimates within the same order of magnitude as LD-N_e_ estimates per generation, albeit with larger CIs. Varying P_crit_ values revealed contrasting effects in the two populations. Specifically, higher values of P_crit_ led to lower N_e_ estimates in KW but caused an upward bias in most estimates for CG (Fig. S7).

**Table 2:** N_e_ estimates obtained by evaluating different cohorts

	Time period	Sample size	Estimate	CI	*N*_e_/*N* point estimate	*N*_e_/*N* median	CI *N*_e_/*N*
KW							
N_e_ generation	2015–2020	226	514	342–949	0.51	0.56	0.34–0.96
N_e_ pooled	2001–2022	380	549	391–861	0.54	0.57	0.39–0.87
N_e_ adjusted	2018 & 2019	52	714	167-inf	0.70	*	0.16-NA*
N	2018	-	1016	935–1105		-	-
CG							
N_e_ generation	2015–2020	233	179	129–266	0.56	0.58	0.39–0.88
N_e_ pooled	2003–2022	325	148	106–215	0.46	0.48	0.32–0.72
N_e_ adjusted	2018 & 2019	83	171	94–538	0.53	0.71	0.30–1.77
N	2018	-	320	264–393		-	-

*for the adjusted N_e_ estimate in KW, the upper CI of N_e_ was infinite. Accordingly, the N_e_/N ratio is reported as the point estimate only, with the lower bound calculated from the lower CI of N_e_ and the upper CI of N. N_e_ generation: 6 consecutive cohorts, N_e_ pooled: all samples, N_e_ adjusted: N_e_ adjusted from N_b_ estimates from two cohorts. N estimates as reported in Edelhoff et al. [20]

### N_e_/N

**Fig. 2:**
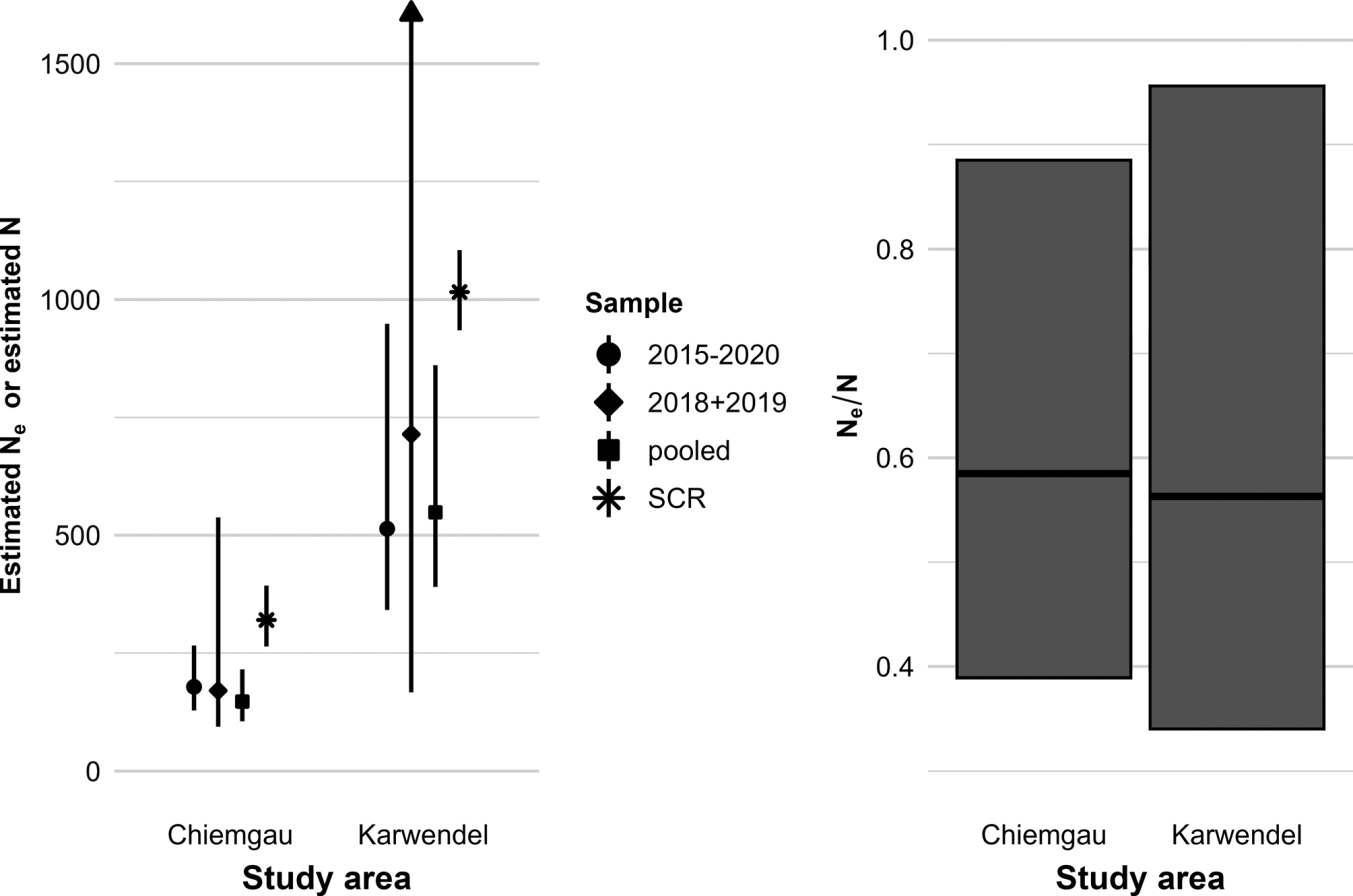
Left: estimated N_e_ values from three different samples (generation sample, two-cohort sample (N_b_) and pooled sample with all individuals) and N estimates from SCR for the study areas Chiemgau and Karwendel. Right: N_e_/N ratio calculated from estimated generation N_e_ and SCR N

In line with our prediction, the median N_e_/N ratio derived from the 2015–2020 generation was slightly greater in CG (0.58) than in KW (0.56, Fig. 2). Combining the credibility intervals of the SCR estimate and confidence intervals for LD-N_e_ resulted in a large 95% uncertainty interval for N_e_/N in both study areas (KW 0.34-96, CG 0.39-88). The distribution of the ratio was moderately asymmetric, with the median exceeding the mean, reflecting uncertainty in N_e_ estimates. The N_e_/N ratios derived from N_b_ estimates ranged within the same order of magnitude but may be less robust due to the large confidence intervals of N_b_ estimates.

### Sensitivity to sample size

**Fig. 3:**
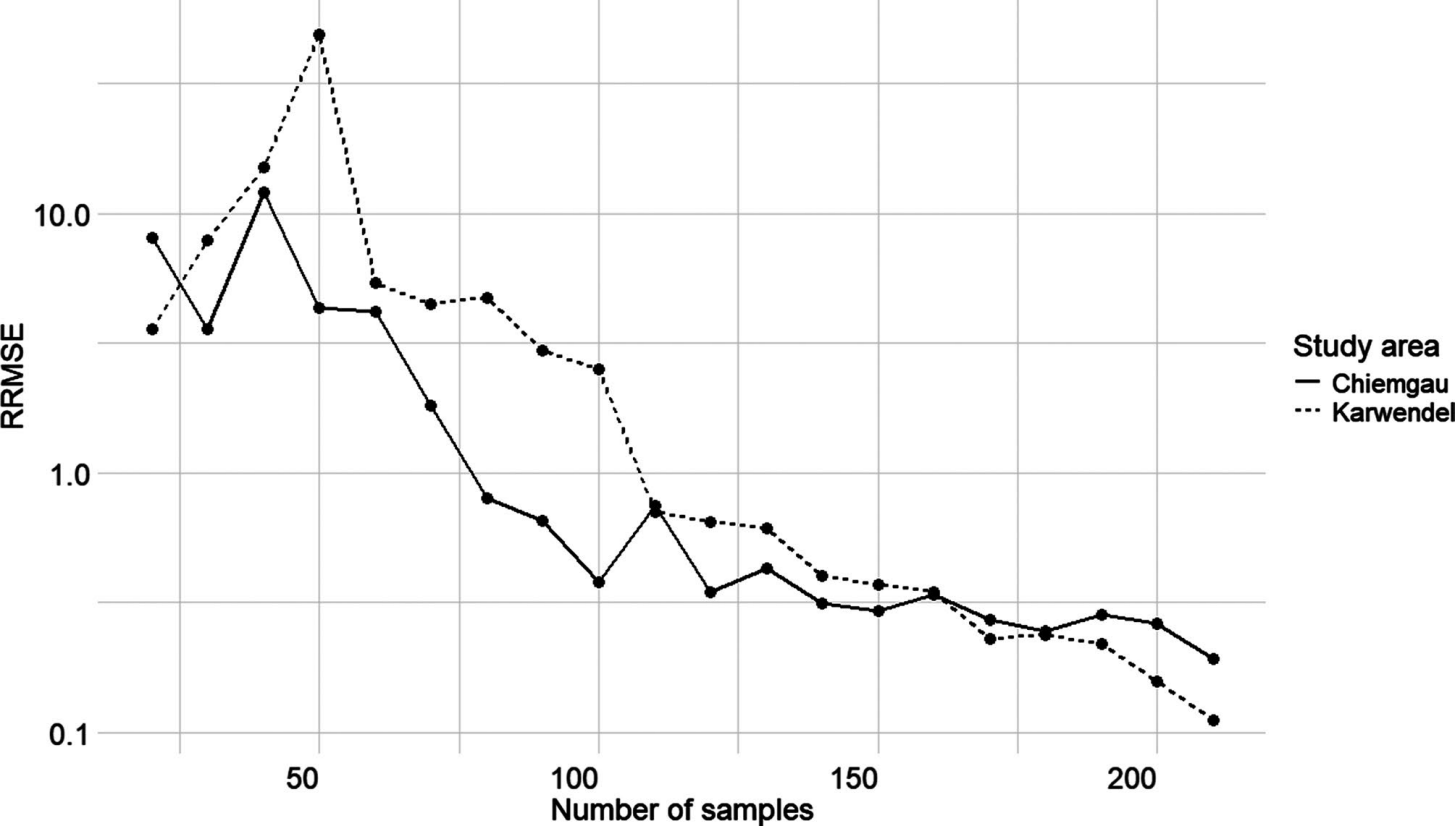
Relative root mean squared error (RRMSE) of N_e_ estimates with varying sample sizes. The N_e_ estimated with the full sample was used as the “true” value. The y-scale was log^10^-transformed to account for the high RRSME with low sample sizes

Small sample sizes caused large confidence intervals and over- or underestimation of N_e_ (relative to the N_e_ calculated from the “full” sample, Fig.). Increasing the sample size led to a decrease in the relative error (RRMSE) when the estimate from the complete sample was used as the “real value”. With low sample sizes (approx. < 150), the relative bias was greater in KW and decreased more slowly than in CG (Fig. 3). Additionally, estimates based on small samples resulted in high percentages of negative estimates and infinite confidence intervals (Fig. S9). This effect was more pronounced in KW compared to CG.

## Discussion

In this study, we took advantage of the rare opportunity to obtain detailed and high-resolution data on both precise estimates of true population size and the genetically derived effective population size of a species of high conservation and management concern in two contrasting Bavarian study areas. This data availability allowed us to compare both estimates. As hypothesized, the difference in N_e_ values was in concordance with the estimated population size differences, resulting in similar N_e_/N ratios in both study areas. However, our results also highlight some important constraints in the applicability of N_e_ as an indicator for conservation purposes, particularly when used as a measure of population size in a conservation context.

In general, our mean N_e_/N values corresponded with those expected from the literature for long-lived mammal species with intermediate ages at maturity and small litter sizes (usually a single offspring per female per year in the case of chamois; [15]). Since our estimates of N refer to the total population size, including an unknown proportion of kids and juveniles, whereas most data in the literature refer to adult population size [50], our N_e_/N ratio is likely biased low. On the basis of life-history data, Waples et al. [75] determined a mean N_e_/N of 0.749 for mammals, a value much larger than the standard ratio of 0.1 often used for conservation purposes. Examples of ungulate species mentioned in the study include bighorn sheep (*Ovis canadensis*, 0.690), red deer (*Cervus elaphus*, 0.946) and Dall’s sheep (*Ovis dalli*, 0.704). Other sources report considerably lower values. Clarke et al. [10] calculated a median N_e_/N of 0.17 for mammal species in a review of studies with empirical, single sample N_e_ estimates. Examples of empirical assessments of ungulates are listed in a large review by Hoban et al. [29], e.g., N_e_/N values of 0.23–0.41 for red deer or 0.42–0.44 for bighorn sheep. These species may, however, exhibit greater individual differences in terms of male mating success than chamois because of their mating system. Chamois have been formerly described to be highly polygynous [39], albeit more recent data hint at moderate polygyny and thus moderate interindividual variability in male reproductive success [6, 14]. Most life-history parameters are not known in detail for chamois. Age- and sex-specific mortality patterns may differ between areas [5], and the degree of variability in reproductive success remains unclear despite several studies on chamois reproductive tactics [12, 14, 16]. Therefore, demographic prediction or modeling of effective population sizes in comparison with genetic values and their relation to values obtained for other species is difficult.

Our estimates of the N_e_/N ratio are slightly higher than the estimates that have been determined for two small, introduced *R. r. rupicapra* populations in Slovakia (0.439 and 0.447), but confidence intervals overlapped [78]. In both the Slovakian study and our study, N refers to estimates of total population size rather than adult population size. However, Slovakian N_e_ estimates were calculated with samples from multiple overlapping generations, and the examined populations have suffered severe bottlenecks, both of which may lead to lower LD-N_e_ estimates.

Contrary to our expectations, despite extensive multiyear genetic sampling, N_e_ estimates displayed large confidence intervals, particularly in the KW study area. Our analyses revealed that confidence intervals become large or that measures become infinite when the sample size is small compared with N_e_. Comprehensive and precise N_e_ estimates for larger populations therefore require extensive sampling effort that may be challenging to achieve for populations of least concern (i.e., with evidence of N_e_ > > 500). Additional effects on local N_e_ stem from high rates of migration or genetic exchange with neighboring populations. While restricting samples to approximately one generation has been proposed as an approximation for generational LD-N_e_ estimates in species with overlapping generations [73, 76], our data indicate that incorporating all available samples may lead to more accurate and precise estimates for larger populations with low S/N_e_. In addition to N_e_, for monitoring over shorter time spans, the effective number of breeders (N_b_) has been proposed as a suitable parameter, especially for species with age structure, i.e., iteroparous species with overlapping generations [76]. However, in our study, the restriction of samples to single cohorts led to a reduction in sample size that caused large CIs. The application of N_b_ for monitoring or as an approach to estimating N_e_ would therefore require targeted and extensive sampling of the specific cohorts used for analysis.

In many studies, uncertainty in N_e_/N estimates is not provided, and confidence intervals are scarce for abundance (N) estimates [50, 71]. Lacking a defined statistical method to assess uncertainty in Ne/N ratios [71], we propagated uncertainty from both Ne and N estimates using a Monte Carlo approach to provide an empirical distribution of the ratio.

We therefore focus mainly on factors that may introduce bias to LD-N_e_ estimates. Many assumptions necessary for LD estimates of effective population size are only rarely met in natural populations [70]. In several studies, the influence of deviations from assumptions of the LD-N_e_ method has been modeled; in most cases, simulated populations have been used [27, 65, 69, 70, 74]. In the following paragraphs, we discuss which of these deviations may apply to the populations in our study areas, how these deviations might influence our N_e_ estimates and whether this might be mitigated by adjusting sampling schemes. Some violations of these assumptions likely affected both study areas to a similar degree, whereas others may have been more pronounced in the Karwendel study area, leading to greater variability and larger confidence intervals in the N_e_ estimates.

### Effects of sampling on the precision of N_e_ estimates

Differences in precision between the study areas may arise from the sampling rate. Relative to the estimated population size, the sampling rate was considerably lower in KW than in CG (for the 2015–2020 generational sample: sample (S)/*N* = 0.73 in CG versus sample (S)/*N* = 0.23 in KW). Consequently, the ratio of the sample size to N_e_ (S/N_e_) was also smaller. While a correction for small sample sizes proposed by England et al. [21] has been implemented in NeEstimator, sample sizes much lower than N_e_ may still influence estimates [27]. Regardless, with sufficient markers (10–20 microsatellites according to Waples and Do [73] and an S/N_e_ ratio of > 0.1, reliable estimates of N_e_ up to 500 or 1,000 individuals should be feasible. Both criteria were fulfilled in our study with 16 microsatellites. While small sample sizes may lead to overestimation of N_e_ [65], other studies also reported a tendency to underestimate N_e_ values when sample sizes were low [35, 36] or resulted in large confidence intervals for N_b_ estimates [4], an effect that was also visible for N_b_ values in the current study.

In general, N_e_ estimation by linkage disequilibrium may be less precise for larger populations (500 individuals or more), as the genetic signal of linkage disequilibrium becomes weaker with increasing population size, and sampling noise will gain importance [41]. Some simulation studies have shown robust estimates for populations with larger N_e_ values [65, 77], and the use of a large number of (genomic) markers can improve precision to some extent [49]; however, accurate estimates for large populations and low S/N_e_ ratios remain difficult [66]. In the future, the use of a large number of SNPs may facilitate more precise estimates of N_e_. Our data revealed an unexpectedly high downward trend for N_e_ estimates with larger P_crit_ values in the KW study area, whereas N_e_ estimates surprisingly were higher with larger P_crit_ values in the GC area. Given the number of samples – in the generation sample much larger than the proposed threshold of 100 samples to use P_crit_ = 0.01 [73] – effects from rare alleles are presumed to be small. It remains unclear whether this may be an actual upward bias or an effect of less data used with smaller P_crit_ values, adding to the effect of small sample size to N_e_ ratio. In general, for larger samples, estimates using P_crit_ = 0.01 are expected to be more precise because more data are used [77].

In addition to sample size, the sampling scheme can influence N_e_ estimates. Under optimal conditions, sampling for genetic N_e_ estimates should be random with respect to spatial aspects, age, sex and relatedness between individuals [76]. In our case, sampling is affected by hunting regulations and quotas, which may introduce bias concerning the sex and age of the sampled individuals as well as their spatial distribution. The sex ratio indeed differed between the SCR estimates and samples obtained from harvested individuals, which may influence N_e_ estimates. Thus, while the availability of samples from hunted individuals of known age offers a great advantage for genetic analyses, potential biases need to be considered when interpreting the results.

### Effects of population structure and migration on local N_e_

Evidence of genetic structure within the study areas was not found with the STRUCTURE software. However, positive F_IS_ values indicate that there may be population structures not accounted for, suggesting that the study areas may be larger than a single genetic neighborhood size [46, 54], i.e., the radius in which mating can be considered random. Continuously distributed populations without pronounced genetic structures between neighboring populations substantially deviate from the assumption of the LD-N_e_ method, and the inclusion of individuals from multiple genetic neighborhoods can introduce a downward bias due to the Wahlund effect [46]. Similarly, migration into the study areas may introduce bias, although LD-N_e_ has been shown to be relatively robust to immigration, providing reliable estimates of local N_e_ unless migration rates are high [71, 74]. Chamois are continuously distributed throughout most of the Bavarian Alps. Thus, multiyear N_e_ estimates likely capture genetic information from a larger area, e.g., through migration or through mating with chamois outside the study area. This might lead to an overestimation of N_e_ compared with abundance estimates, which only contain individuals present in the study area during the time frame of sampling (in our case, approximately three weeks; see [20]). Regardless, LD-N_e_ has been shown to be relatively robust to immigration, providing reliable estimates of local N_e_ [74].

Finally, the estimated ratio applies to local populations; hence, it may differ from the metapopulation N_e_/N ratio [10]. The N_e_ > 500 criterion generally refers to the metapopulation size rather than to local populations [10, 71]. However, the metapopulation N_e_ is highly complex, particularly in species with continuous distribution [33, 44].

### Influences of management on N_e_ and N_e_/N

The large confidence intervals of the N_e_/N ratio in our study prevent conclusions from being drawn from supposedly small differences in the N_e_/N ratio between study areas. Different hunting regimes may not only influence the age or sex distribution of samples used for N_e_ estimates as described above but also potentially lead to altered age structure, sex ratios or life-history strategies and can thus affect the effective population size itself [2, 62]. In particular, the annual rate of adult mortality has been shown to affect N_e_ and the N_e_/N ratio [69]; therefore, differences or changes in adult mortality are expected to impact N_e_. In hunted chamois populations, both female and male chamois are assumed to adopt a faster life history [5], which in turn may influence the effective population size [47, 75]. With a multitude of contributing factors, it is difficult to predict to what extent and in which direction the harvest regimes referring to different age classes influence the effective population size without detailed data on population age structure and fecundity. Assessing and monitoring N_e_ and the N_e_/N ratio on a larger spatial scale and over longer time periods, ideally combined with monitoring of life-history parameters, would facilitate deeper insights into the effects of hunting regimes on chamois populations.

## Conclusion

Despite the large confidence intervals, the mean N_e_/N ratio was stable across the study areas. Although the inclusion of a larger spatial scale would be desirable, our data allow for rough comparisons of local N_e_ and N and a useful range of N_e_/N for conservation assessments. Species-specific estimates of the N_e_/N ratio, as calculated in our study, facilitate more precise assessments of N_e_ for populations without available genetic data. However, the large confidence intervals show that even with extensive data, accurately estimating the N_e_/N ratio remains challenging, highlighting potential limitations for their use in population monitoring and management.

## Supplementary Information

Below is the link to the electronic supplementary material.

Supplementary Material 1

## Data Availability

The datasets generated and analyzed as part of this study are available in the Zenodo repository (10.5281/zenodo.17945015).
