# Supplementary material for: Effective population size and Ne/N ratio for two populations of Northern chamois (Rupicapra rupicapra) on the edge of the species’ distribution range
Source: BMC Ecol Evol. 2026 Jun 26;26:53. doi: 10.1186/s12862-026-02543-7 (PMC13310441; doi:10.1186/s12862-026-02543-7)
Supplement: Supplementary file 1 — Supplementary Material 1 [file 12862_2026_2543_MOESM1_ESM.docx]

**Supplementary information**

Effective population size and N_e_/N ratio for two populations of Northern chamois (*Rupicapra rupicapra*) on the edge of the species’ distribution range

Journal: Conservation Genetics

Author names: Susanne Jacobs, Hendrik Edelhoff, Cornelia Ebert, Wibke Peters

Corresponding author: Susanne Jacobs, [susanne.jacobs@lwf.bayern.de](mailto:susanne.jacobs@lwf.bayern.de)

Affiliation: Wildlife Biology and Management Research Unit, Bavarian State Institute of Forestry, Freising, Germany

**Supplementary methods**

Genetic structure within the population under study as well as the presence of migrants from other, differentiated populations can influence measures of LD-Ne (Waples and England 2011). Thus, we analyzed all samples with the software STRUCTURE (Pritchard et al. 2000) to identify possible population structure within and between the study areas. We used the admixture model with correlated allele frequencies and 100,000 runs after a burn-in of 100,000 for the between-study area analysis and 200,000 runs after a burnin of 100,000 for the presumingly more subtle structure within study that may take longer for the analysis to converge. STRUCTURE results were analyzed with the pophelper package (Francis 2017) using the Evanno method (Evanno et al. 2005). STRUCTURE analyses were repeated for each study area individually to check for genetic structure within the areas.

**Supplementary Tables**

**Table S1.** Microsatellites used for individual identification of alpine chamois.

| **Locus** | **Size range (bp)** | **Multiplex** | **Reference** |
| --- | --- | --- | --- |
| **HEL1** | 112 - 132 | A | Cassar et al. 2007 |
| **INRA36** | 176 - 188 | D | Pérez et al. 2000 |
| **OARFCB304** | 128 - 154 | B | Soglia et al. 2005 |
| **BM203** | 214 - 240 | B | Cassar et al. 2007 |
| **BM848** | 218 - 234 | B | Cassar et al. 2007 |
| **BMC1009** | 280 - 320 | A | Cassar et al. 2007 |
| **ETH225** | 134 - 160 | A | Pérez et al. 2000 |
| **TGLA53** | 120 - 200 | C | Zemanová et al. 2011 |
| **CSRD247** | 195 - 245 | C | Soglia et al. 2005 |
| **SR-CRSP08** | 220 - 255 | C | Pérez et al. 2000 |
| **BM1818** | 220 - 300 | C | Cassar et al. 2007 |
| **ILST30** | 120 - 200 | D | Cassar et al. 2007 |
| **KCNA44** | 190 - 260 | D | Cassar et al. 2007 |
| **BM4505** | 210 - 300 | D | Cassar et al. 2007 |
| **BM1329** | 145 - 190 | B | Soglia et al. 2005 |
| **BM1258** | 80 - 160 | B | Soglia et al. 2005 |

**Table S2.** Results of test for Hardy-Weinberg-Equilibrium for individual microsatellite loci.

| **Study area** | **Karwendel** | | | | **Chiemgau** | | | |
| --- | --- | --- | --- | --- | --- | --- | --- | --- |
| **Locus** | **chi²** | **df** | **p chi²** | **p exact** | **chi²** | **df** | **p chi²** | **p exact** |
| **BM1258** | 76.12 | 55 | 0.03 | 0.2 | 87.67 | 66 | 0.04 | 0.14 |
| **BM1329** | 15.97 | 15 | 0.38 | 0.37 | 17.8 | 21 | 0.66 | 0.51 |
| **BM1818** | 51.61 | 66 | 0.9 | 0.72 | 428.92 | 78 | 0 | 0.16 |
| **BM203** | 55.88 | 66 | 0.81 | 0.37 | 79.9 | 66 | 0.12 | 0.09 |
| **BM4505** | 396.66 | 28 | 0 | 0.05 | 9.87 | 21 | 0.98 | 0.73 |
| **BM848** | 9.08 | 6 | 0.17 | 0.16 | 14.16 | 10 | 0.17 | 0.13 |
| **BMC1009** | 15.5 | 28 | 0.97 | 0.79 | 1.54 | 3 | 0.67 | 0.46 |
| **CRSP08** | 58.75 | 66 | 0.72 | 0.16 | 39.93 | 55 | 0.94 | 0.44 |
| **CSRD247** | 67.12 | 78 | 0.81 | 0.79 | 24.32 | 28 | 0.66 | 0.15 |
| **ETH225** | 6.74 | 15 | 0.96 | 0.94 | 2.3 | 6 | 0.89 | 0.82 |
| **HEL1** | 31.34 | 28 | 0.3 | 0.5 | 61.11 | 28 | 0 | 0.03 |
| **ILST30** | 14.74 | 15 | 0.47 | 0.52 | 19.27 | 15 | 0.2 | 0.26 |
| **INRA36** | 12.84 | 15 | 0.61 | 0.51 | 114.78 | 15 | 0 | 0 |
| **KCNA44** | 20.42 | 28 | 0.85 | 0.59 | 34.76 | 36 | 0.53 | 0.58 |
| **OARFCB304** | 37.43 | 28 | 0.11 | 0.03 | 41.32 | 10 | 0 | 0 |
| **TGLA53** | 6.4 | 10 | 0.78 | 0.62 | 10.16 | 6 | 0.12 | 0.12 |

**Table S3.** Results for observed heterozygosity (Ho), expected heterozygosity (He), inbreeding coefficient (FIS) and allelic richness (Ar) for individual microsatellite loci.

| **Study area** | **Karwendel** | | | | **Chiemgau** | | | |
| --- | --- | --- | --- | --- | --- | --- | --- | --- |
| **Locus** | **Ho** | **He** | **FIS** | **Ar** | **Ho** | **He** | **FIS** | **Ar** |
| **BM1258** | 0.829 | 0.839 | 0.012 | 10.975 | 0.825 | 0.847 | 0.026 | 12 |
| **BM1329** | 0.803 | 0.802 | -0.001 | 6 | 0.809 | 0.778 | -0.04 | 7 |
| **BM1818** | 0.78 | 0.785 | 0.007 | 11.845 | 0.781 | 0.785 | 0.005 | 13 |
| **BM203** | 0.754 | 0.792 | 0.047 | 11.852 | 0.694 | 0.7 | 0.008 | 11.987 |
| **BM4505** | 0.582 | 0.617 | 0.058 | 7.793 | 0.545 | 0.555 | 0.018 | 7 |
| **BM848** | 0.587 | 0.588 | 0.002 | 4 | 0.634 | 0.629 | -0.007 | 5 |
| **BMC1009** | 0.532 | 0.553 | 0.039 | 7.817 | 0.36 | 0.383 | 0.061 | 2.985 |
| **CRSP08** | 0.828 | 0.829 | 0.001 | 11.67 | 0.551 | 0.546 | -0.009 | 10.969 |
| **CSRD247** | 0.746 | 0.722 | -0.033 | 12.977 | 0.677 | 0.684 | 0.01 | 7.969 |
| **ETH225** | 0.442 | 0.42 | -0.053 | 5.999 | 0.394 | 0.387 | -0.017 | 4 |
| **HEL1** | 0.749 | 0.784 | 0.044 | 7.82 | 0.742 | 0.766 | 0.032 | 7.985 |
| **ILST30** | 0.618 | 0.648 | 0.045 | 6 | 0.705 | 0.724 | 0.026 | 6 |
| **INRA36** | 0.646 | 0.655 | 0.015 | 6 | 0.622 | 0.661 | 0.059 | 6 |
| **KCNA44** | 0.778 | 0.79 | 0.014 | 7.976 | 0.717 | 0.712 | -0.007 | 8.985 |
| **OARFCB304** | 0.702 | 0.746 | 0.059 | 7.844 | 0.631 | 0.675 | 0.065 | 5 |
| **TGLA53** | 0.55 | 0.587 | 0.063 | 5 | 0.425 | 0.423 | -0.004 | 4 |

**Table S4.** NeEstimator results for effective population size (N_e_) for both study areas (CG = Chiemgau, KW = Karwendel) with pseudo-jacknife confidence intervals. Sample = cohorts included in the analysis (pooled = all samples, marker = without markers not in HWE (CG) or with evidence for null alleles (KW)); migrants: without individuals identified as potential migrants; N = number of samples; Ind Alleles: number of independent alleles; Type = parameter (N_e_ or N_e_adj = N_e_ calculated from N_b_); pCrit = critical value for allele frequency as exclusion criterion for rare alleles (1/2S = 1/ 2 * sample size)

| Study area | Sample | N | Ind Alleles | Type | N_e_ value | lower jacknife | upper jacknife | pCrit |
| --- | --- | --- | --- | --- | --- | --- | --- | --- |
| CG | 2015-2020 | 233 | 1791 | N_e_ | 198.5 | 140.3 | 306 | 0.05 |
| CG | 2015-2020 | 233 | 2759 | N_e_ | 183.4 | 135.3 | 264 | 0.02 |
| **CG** | **2015-2020** | **233** | **3672** | **N_e_** | **178.8** | **129** | **265.6** | **0.01** |
| CG | 2015-2020 | 233 | 4537 | N_e_ | 140.3 | 101.3 | 205.6 | 1/2S |
| CG | pooled | 325 | 1850 | N_e_ | 168.7 | 131.9 | 221.8 | 0.05 |
| CG | pooled | 325 | 2979 | N_e_ | 162 | 130.5 | 205.1 | 0.02 |
| **CG** | **pooled** | **325** | **3758** | **N_e_** | **147.6** | **105.9** | **215.1** | **0.01** |
| CG | pooled | 325 | 4616 | N_e_ | 137.3 | 107.7 | 179 | 1/2S |
| CG | 2018+2019 | 83 | 1575 | N_e_adj | 155.6 | 86.5 | 464.6 | 0.05 |
| **CG** | **2018+2019** | **83** | **2750** | **N_e_adj** | **170.8** | **94.4** | **537.5** | **0.02** |
| CG | 2018+2019 | 83 | 3346 | N_e_adj | 166.5 | 104.7 | 345.0 | 0.01 |
| CG | 2018+2019 | 83 | 3346 | N_e_adj | 166.5 | 104.7 | 345.0 | 1/2S |
| CG | 2015-2020, marker | 233 | 2937 | N_e_ | 191.1 | 140.9 | 275.7 | 0.01 |
| CG | pooled, marker | 325 | 3009 | N_e_ | 159.2 | 127.6 | 202.8 | 0.01 |
| CG | 2015-2020, migrants | 231 | 3516 | N_e_ | 195.6 | 144.3 | 282.8 | 0.01 |
| CG | pooled, migrants | 322 | 3498 | N_e_ | 156.1 | 114.9 | 220.9 | 0.01 |
| KW | 2015-2020 | 226 | 2580 | N_e_ | 448 | 228.3 | 2181.5 | 0.05 |
| KW | 2015-2020 | 226 | 4318 | N_e_ | 474.2 | 321.2 | 836.8 | 0.02 |
| **KW** | **2015-2020** | **226** | **5116** | **N_e_** | **513.6** | **341.6** | **948.5** | **0.01** |
| KW | 2015-2020 | 226 | 5716 | N_e_ | 553.1 | 376.4 | 974.5 | 1/2S |
| KW | pooled | 380 | 2607 | N_e_ | 523.7 | 352.2 | 912.1 | 0.05 |
| KW | pooled | 380 | 4382 | N_e_ | 507.3 | 377.9 | 735.6 | 0.02 |
| **KW** | **pooled** | **380** | **5128** | **N_e_** | **548.5** | **390.6** | **860.5** | **0.01** |
| KW | pooled | 380 | 6238 | N_e_ | 707.8 | 498.8 | 1145.8 | 1/2S |
| KW | 2018+2019 | 52 | 1964 | N_e_adj | 7254.0 | 161.7 | Inf | 0.05 |
| **KW** | **2018+2019** | **52** | **3506** | **N_e_adj** | **714.2** | **167.4** | **Inf** | **0.02** |
| KW | 2018+2019 | 52 | 4284 | N_e_adj | 993.7 | 205.7 | Inf | 0.01 |
| KW | 2018+2019 | 52 | 4166 | N_e_adj | 1511.1 | 221.7 | Inf | 1/2S |
| KW | 2015-2020, marker | 226 | 3797 | N_e_ | 524.1 | 326.5 | 1145.2 | 0.01 |
| KW | pooled, marker | 380 | 3809 | N_e_ | 552.4 | 380.7 | 921.1 | 0.01 |
| KW | 2015-2020, migrants | 225 | 5116 | N_e_ | 506.7 | 336.9 | 935.8 | 0.01 |
| KW | pooled, migrants | 374 | 5128 | N_e_ | 552.3 | 391.4 | 874.9 | 0.01 |

**Supplementary Figures**

**
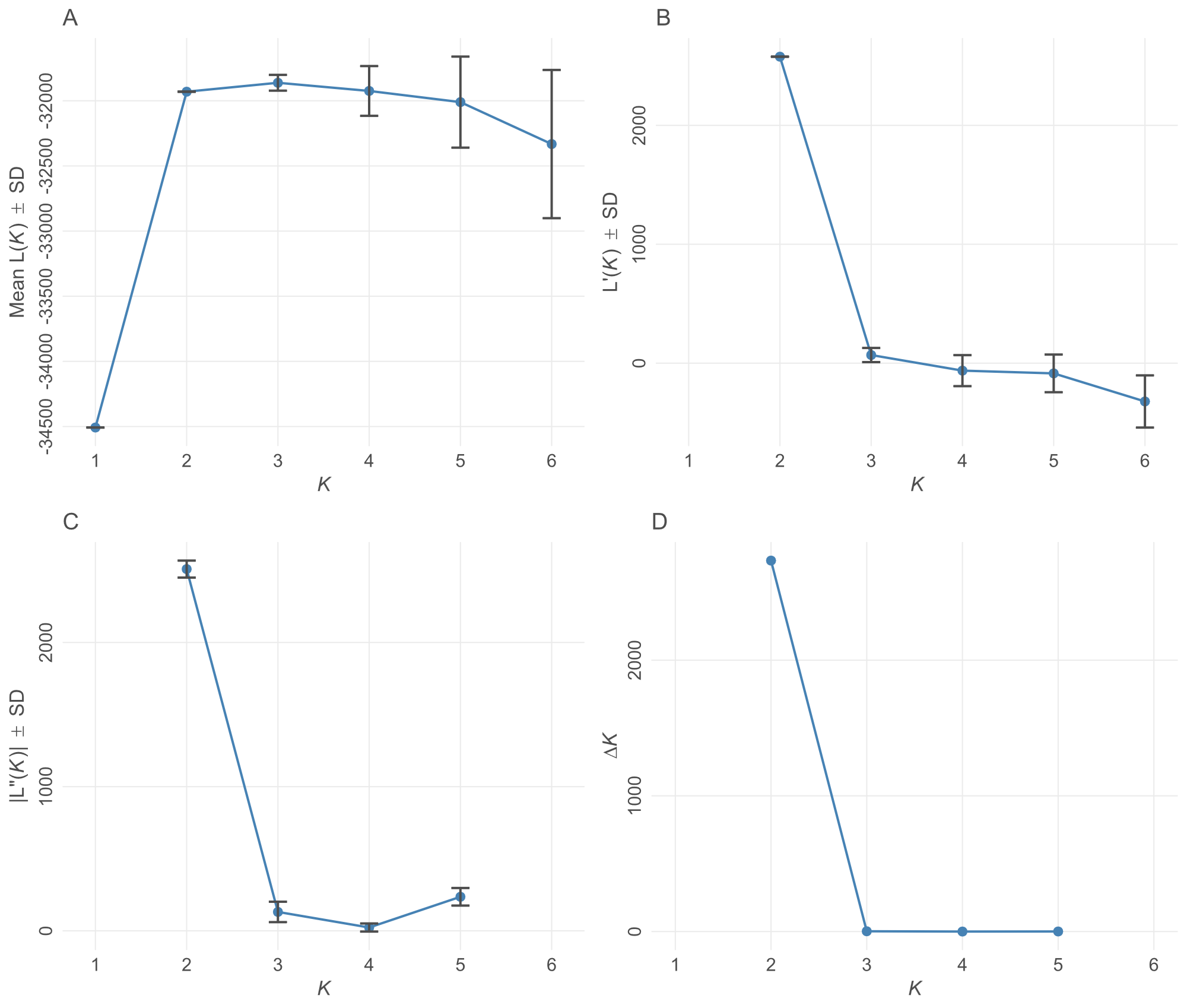
**

**Fig. S1.** STRUCTURE results for population structure between study areas analyzed with the R package pophelper. A: Mean ln probability of one (K = 1) to six (K = 6) genetic clusters and standard deviation. B: First derivative of mean ln probability of one (K = 1) to six (K = 6) genetic clusters and standard deviation. C: Second derivative of mean ln probability of one (K = 1) to six (K = 6) genetic clusters and standard deviation. D: deltaK


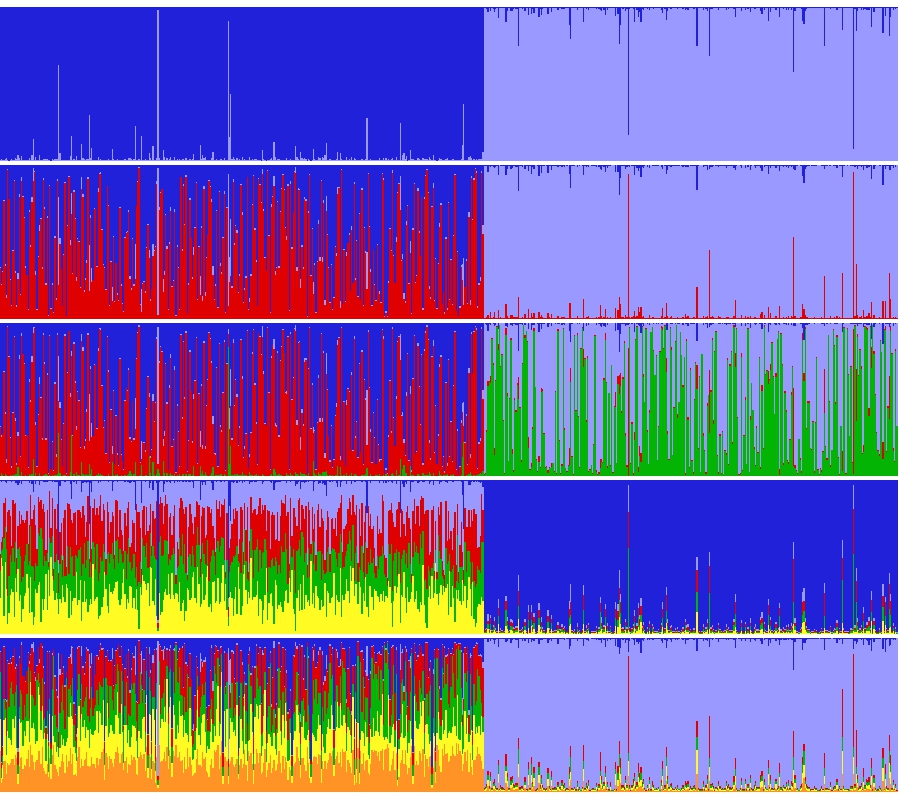


**Fig. S2.** Q matrices for K = 2 to K = 5 from STRUCTURE results for population structure between the study areas analyzed with the R package pophelper

**
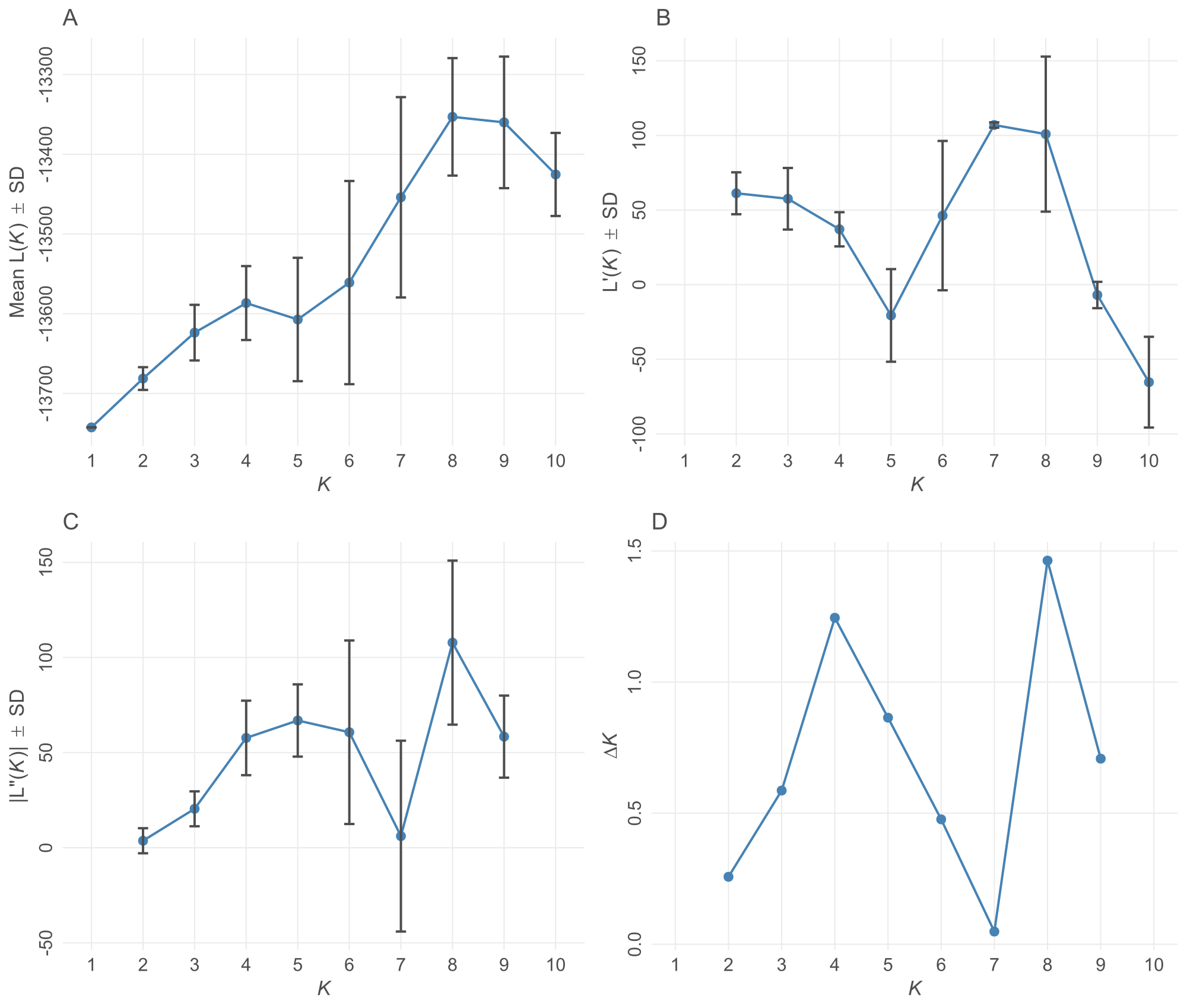
**

**Fig. S3.** STRUCTURE results for the study area Chiemgau analyzed with the R package pophelper. A: Mean ln probability of one (K = 1) to ten (K = 10) genetic clusters and standard deviation. B: First derivative of mean ln probability of one (K = 1) to ten (K = 10) genetic clusters and standard deviation. C: Second derivative of mean ln probability of one (K = 1) to ten (K = 10) genetic clusters and standard deviation. D: deltaK


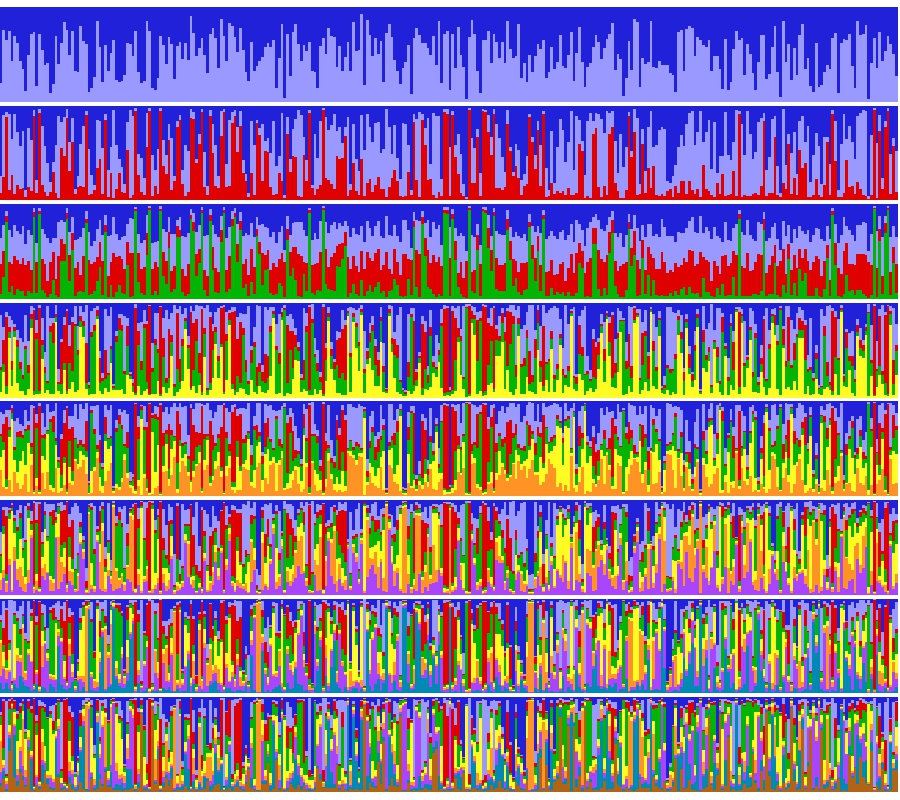


**Fig. S4.** Q matrices for K = 2 to K = 8 from STRUCTURE results for the study area Chiemgau analyzed with the R package pophelper


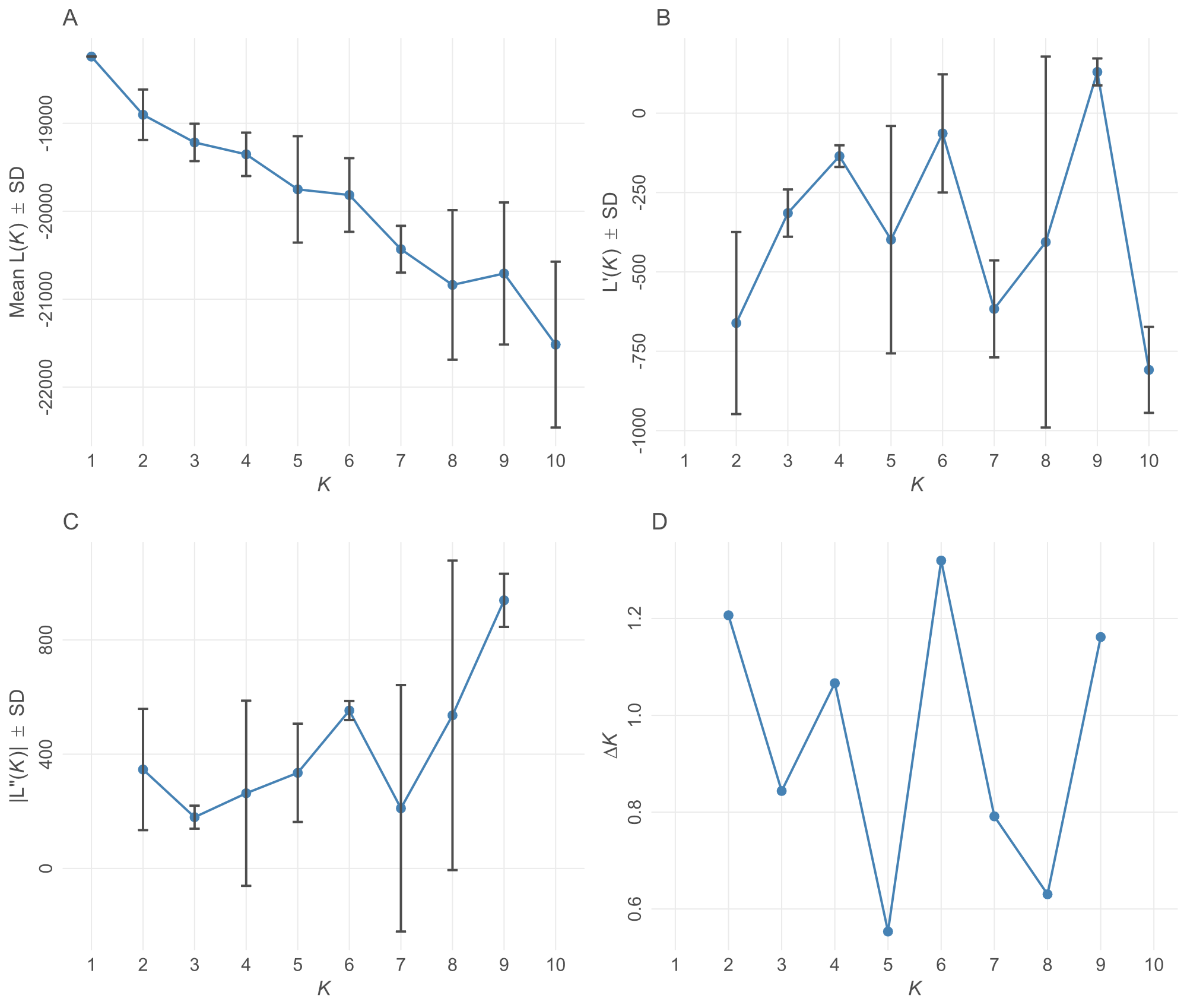
**Fig. S5.** STRUCTURE results for the study area Karwendel analyzed with the R package pophelper. A: Mean ln probability of one (K = 1) to ten (K = 10) genetic clusters and standard deviation. B: First derivative of mean ln probability of one (K = 1) to ten (K = 10) genetic clusters and standard deviation. C: Second derivative of mean ln probability of one (K = 1) to ten (K = 10) genetic clusters and standard deviation. D: deltaK


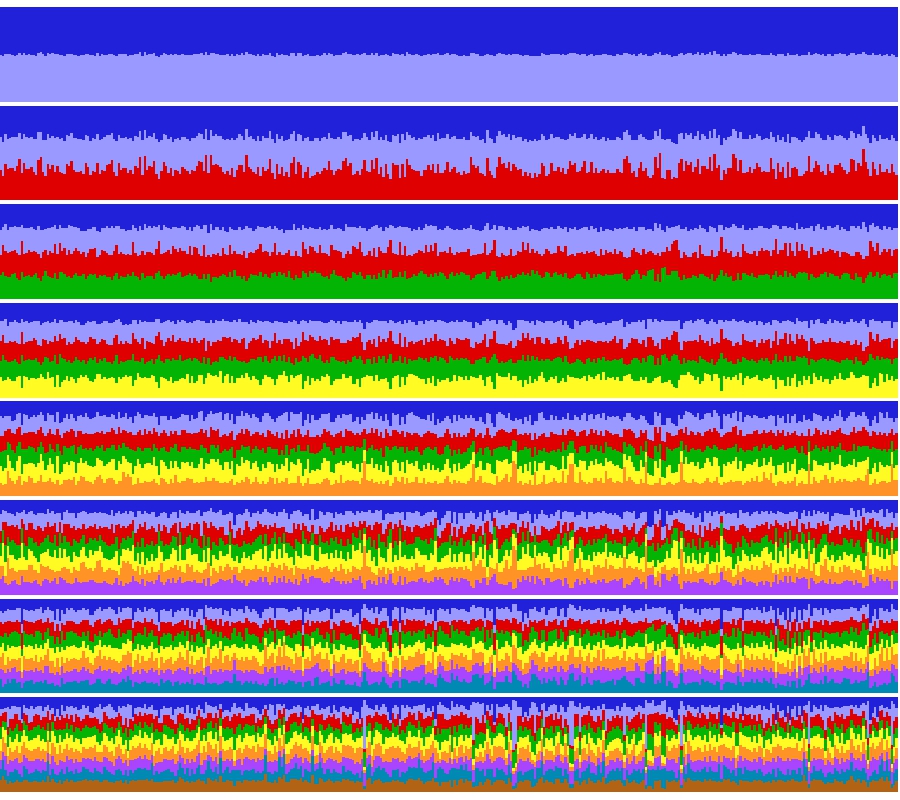
**Fig. S6.** Q matrices for K = 2 to K = 8 from STRUCTURE results for the study area Karwendel analyzed with the R package pophelper

**Fig. S7.** Results for different P_crit_ values for the study areas Chiemgau and Karwendel.


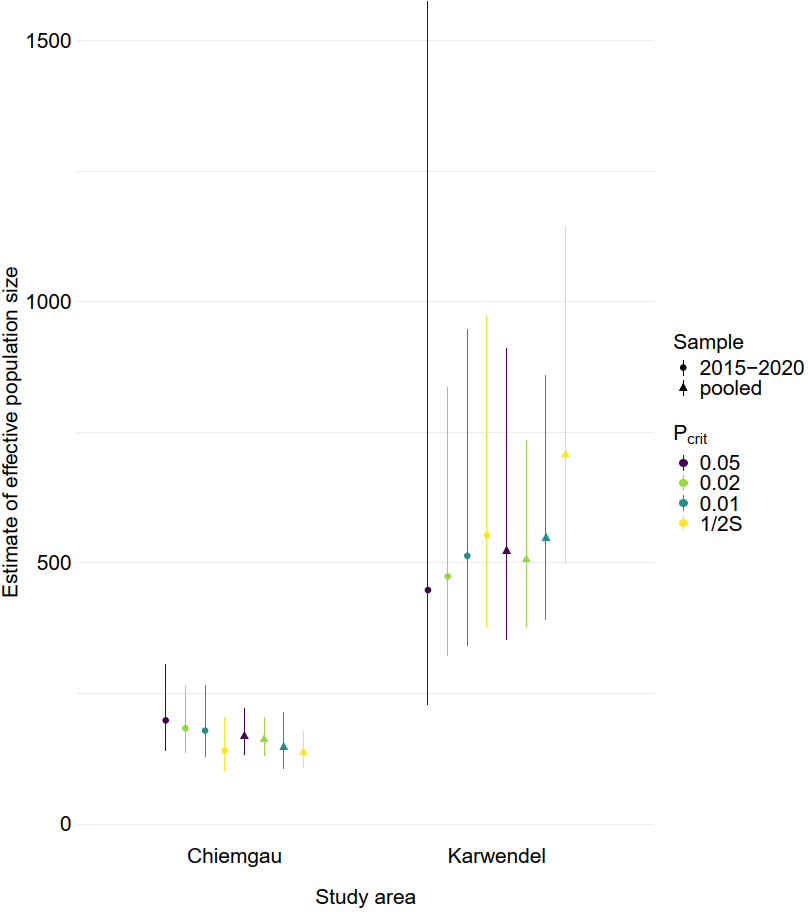


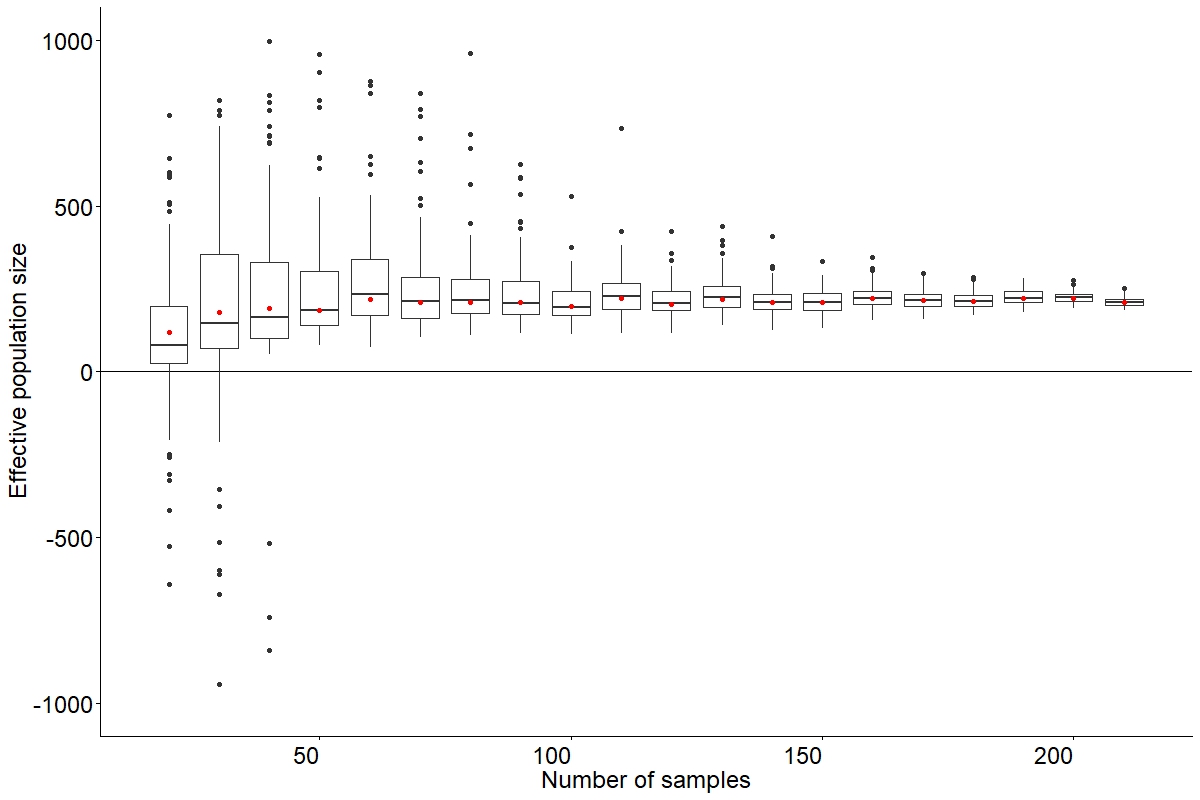


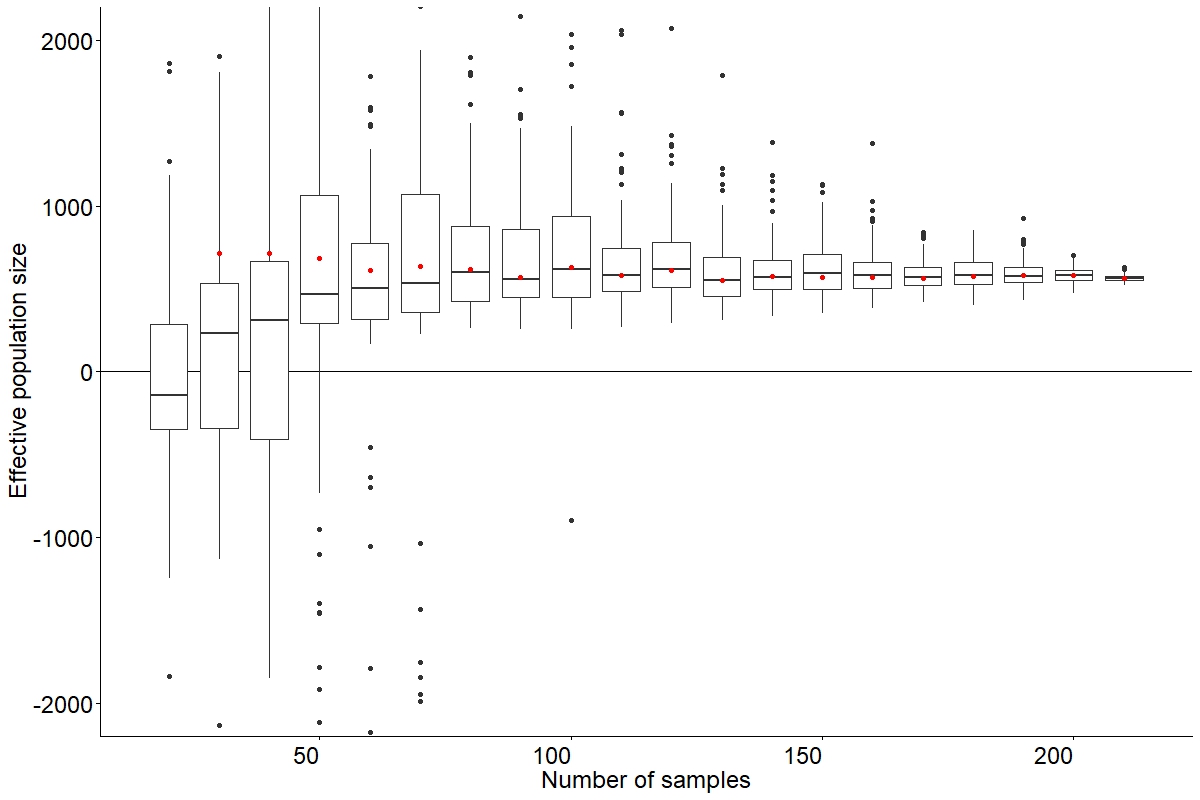


**Fig S8.** Boxplots of N_e_ estimates obtained from subsampling (100 repetitions per sample size category) for the study area Chiemgau (top) and Karwendel (bottom). Red dots show the harmonic mean of N_e_ estimates. Different scales apply for the study areas due to the difference in N_e_. Scales are trimmed to approx. 4N_e_ for reasons of clarity.


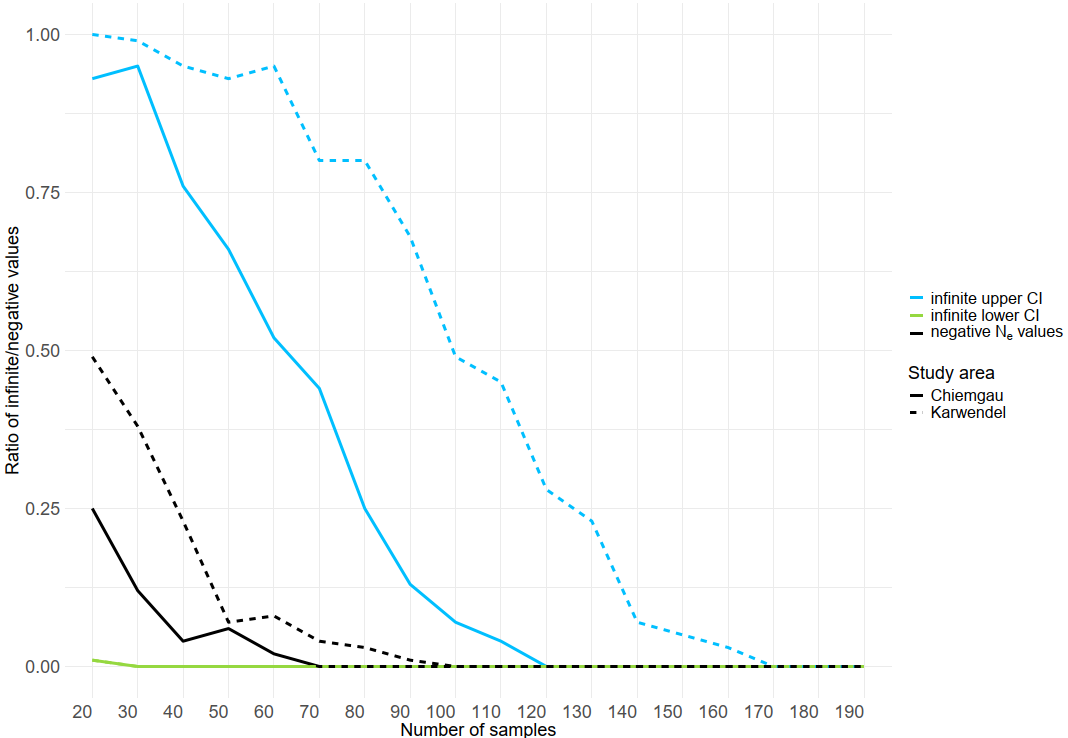


**Fig. S9.** Ratio of infinite upper and lower confidence intervals and negative N_e_ values in relation to sample size.
